# Association between nutritional risk and CRP levels in patients with acute exacerbation of chronic obstructive pulmonary disease

**DOI:** 10.3389/fmed.2025.1611981

**Published:** 2025-06-24

**Authors:** Peihong Liang, Jun Wu

**Affiliations:** ^1^Guangdong Medical University (GDMU), Zhanjiang, Guangdong Province, China; ^2^Clinical Nutrition Department, Maoming People’s Hospital, Guangdong Province, China; ^3^Department of Respiratory and Critical Care Medicine, Affiliated Hospital of Guangdong Medical University, Zhanjiang, Guangdong Province, China

**Keywords:** chronic obstructive pulmonary disease, AECOPD, C-reactive protein, nutritional risk, retrospective study

## Abstract

**Background:**

Patients with acute exacerbation of COPD frequently experience malnutrition, while the quantitative relationship between CRP and nutritional risk remains undefined. This study is the first to investigate this association.

**Methods:**

This retrospective cohort study analyzed 313 hospitalized patients diagnosed with acute exacerbation of chronic obstructive pulmonary disease (AECOPD). Participants were stratified into a nutritional risk group (*n* = 55) and a non-risk group (*n* = 258) using the NRS-2002 screening tool. Clinical data were analyzed via Wilcoxon rank-sum tests, binary logistic regression, and restricted cubic splines (RCS) to model the nonlinear CRP-nutritional risk relationship. Feature importance was further validated through SHAP (SHapley Additive exPlanations) machine learning interpretability frameworks.

**Results:**

The nutritional risk was significantly increased in high CRP group; RCS curve showed that CRP was positively correlated with risk; SHAP model showed that high CRP eigenvalue may be associated with increased nutritional risk.

**Conclusion:**

This study highlights the significant correlation between CRP levels and nutritional risk in patients with AECOPD, providing evidence for nutritional risk assessment and early intervention in patients with AECOPD.

## 1 Introduction

Chronic obstructive pulmonary disease (COPD) is a chronic respiratory disease characterized by airflow limitation, primarily caused by long-term smoking and air pollution. This disease significantly impacts the quality of life of patients, leading to high mortality rates, increased medical costs, and a substantial economic burden on patients, families, and society. According to global epidemiological data, COPD affects 8 to 10% or more of the population over 40 years of age, making it one of the leading causes of mortality worldwide (1). COPD patients often experience long-term chronic dyspnea and recurrent lung infections, which together result in malnutrition (2), progressive weight loss, clinically referred to as “pulmonary cachexia syndrome.” Particularly in severe cases of COPD, protein-energy malnutrition is common and is associated with increased mortality and morbidity, emphasizing the necessity for early nutritional assessment and intervention (3).

In patients with AECOPD, the risk of malnutrition is further aggravated by shortened meal times and reduced food intake due to breathing difficulties. However, existing nutritional risk screening tools, such as the NRS-2002 score (4), may not accurately reflect this situation because the data are derived from medical records, and the dietary details of some patients may be missing, leading to an underestimation of nutritional risk assessment (5). In addition, inflammatory factors such as IL-6 and TNF-*α* can promote muscle proteolysis (6), inhibit appetite, and then lead to malnutrition. C-reactive protein (CRP), as a sensitive indicator of inflammation, may be intrinsically associated with nutritional risks. Although studies have focused on the association between nutritional risk and respiratory diseases, the quantitative relationship between CRP levels and nutritional risk in patients with AECOPD is not clear, and large-sample studies are lacking. Studies have suggested that the mortality rate of malnourished COPD patients is significantly increased. This study used SGA to assess nutritional status (7), but did not pay attention to the relationship between CRP and nutritional risks. This suggests that we need to further explore the role of CRP in investigating nutritional risk in patients with AECOPD.

## 2 Materials and methods

### 2.1 Object of study

#### 2.1.1 Inclusion criteria

1. Met diagnostic criteria for acute exacerbation of chronic obstructive pulmonary disease (AECOPD) as defined by the 2023 Global Initiative for Chronic Obstructive Lung Disease (GOLD) guidelines.
2. The clinical manifestations are predominantly characterized by exacerbation of respiratory symptoms, typically including worsening cough, increased sputum volume, or aggravated dyspnea, which may be accompanied by purulent sputum production, hemoptysis, or low-grade fever in some cases. The stable phase is defined as the maintenance of baseline symptom patterns with respect to cough frequency, sputum characteristics, and dyspnea intensity, where patients demonstrate either sustained clinical stability or minimal symptomatic fluctuation without meeting criteria for acute deterioration.
3. The patient was conscious.
4. Select patients aged 18 years or older.

#### 2.1.2 Exclusion criteria

1. Concomitant severe bronchiectasis, bronchial asthma, or other respiratory diseases.
2. Severe hepatic or renal dysfunction.
3. Immune system disorders with long-term immunosuppressant use.
4. Cognitive impairment, psychiatric disorders, coma, or related neurological conditions.
5. Recent enteral or parenteral nutritional support therapy.

### 2.2 Baseline characteristics

This retrospective study enrolled 313 patients diagnosed with AECOPD, hospitalized in the Department of Respiratory Medicine at our institution between January 2023 and December 2024. Demographic and clinical parameters—including gender, age, smoking status, alcohol consumption, and body mass index (BMI)—were systematically collected. Subsequent statistical analyses were conducted to evaluate these variables. The study protocol received ethical approval from the Institutional Review Board (Ethics Approval Number: PJ2025MI-K032-01).

### 2.3 Statistical methods

All data processing and analyses were performed using R software (version 4.1.1). Non-normally distributed continuous variables were described as median with interquartile range (IQR). Weighted binary logistic regression analyzed associations between nutritional risk groups and CRP, which was incorporated as both continuous and categorical variables (quartiles: Q1-Q4, with Q1 as reference). Three models were constructed: Model 1 (unadjusted), Model 2 (adjusted for sex, age, smoking, alcohol, SBP, DBP), and Model 3 (further adjusted for diabetes, hypertension, kidney disease, cardiovascular/cerebrovascular diseases, PO2Δ).

Machine learning utilized XGBoost with SHAP interpretation. SHAP summary plots quantified variable contributions (BMI, CRP, hemoglobin, PO2Δ), while dependency plots revealed feature-prediction relationships. Interaction analyses assessed subgroup-CRP interplay. Weighted percentages for categorical variables were analyzed via Rao-Scott χ^2^ tests; continuous variables used Wilcoxon rank-sum tests. False discovery rate (FDR)-adjusted *p*-values and two-sided statistical tests were applied, with significance at *p* < 0.05.

## 3 Results

### 3.1 Baseline characteristics

**Figure 1:**
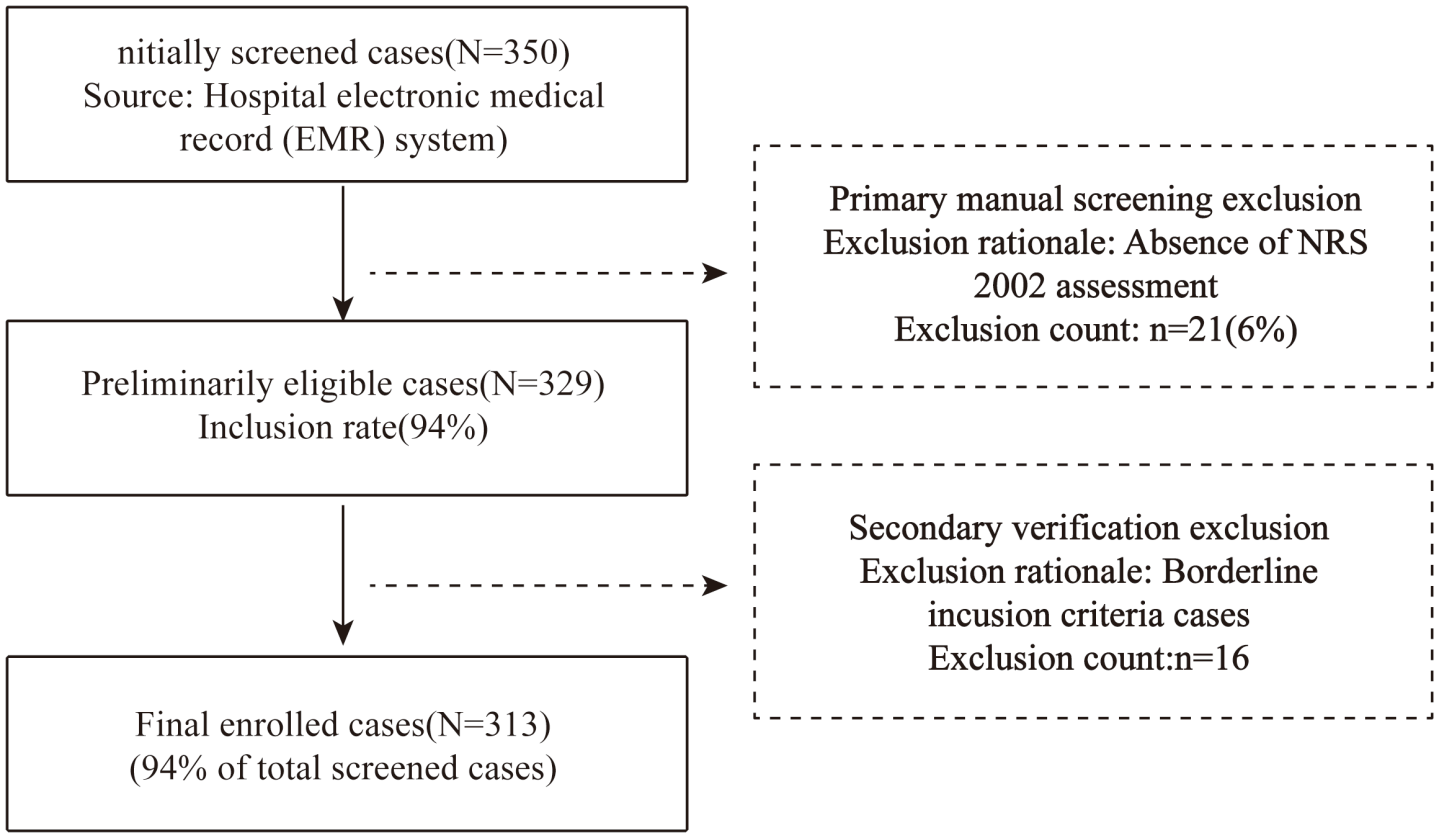
Flow chart for the selection of participants in the study.

The study initially identified 350 eligible cases through hospital electronic medical record (EMR) screening. Following primary manual exclusion of 21 cases lacking Nutritional Risk Screening 2002 (NRS 2002) assessments, 329 candidates remained. Secondary review excluded 16 borderline cases, yielding a final cohort of 313 patients (203 males [65%], 110 females [35%]) aged 41–100 years (mean 74 ± 10). Stratified by nutritional risk status into non-risk (*n* = 258) and risk (*n* = 55) groups, all participants received standardized therapies including anti-infectives, bronchodilators, and expectorants (Figure 1).

There was no statistically significant difference in gender distribution between the two groups (*p* = 0.92). The mean age of the overall patient population was 74 years (standard deviation 10), with the no nutritional risk group and the nutritional risk group having mean ages of 74 (standard deviation 10) and 75 (standard deviation 11) years, respectively, showing no statistically significant difference (*p* = 0.47).

Regarding routine blood test indicators, most parameters such as white blood cell count (WBC), neutrophils, and lymphocytes showed no statistically significant differences between the two groups. For example, the total WBC was 7.7 (6.1, 9.9) × 10^9/L, with the two groups recording 7.7 (6.1, 9.9) × 10^9/L and 8.4 (5.8, 10.5) × 10^9/L, respectively (*p* = 0.95). However, hemoglobin levels differed significantly between the two groups, with a median of 131 (117, 142) g/L in the no nutritional risk group and 123 (106, 133) g/L in the nutritional risk group (*p* = 0.005). CRP levels also showed a significant difference, with the no nutritional risk group at 3 (1, 5) mg/L and the nutritional risk group at 8 (2, 22) mg/L (*p* = 0.004). Although prealbumin (PAB) and albumin (Alb) showed a trend toward difference, they did not reach statistical significance (*p* = 0.065 and *p* = 0.29, respectively).

Other indicators: BMI showed a highly statistically significant difference between the two groups (*p* < 0.001), with the no nutritional risk group at 21.3 (19.2, 23.6) kg/m^2^ and the nutritional risk group at 16.9 (15.6, 17.7) kg/m^2^. Smokers accounted for 73% (228 cases) of the total population, with proportions of 72 and 75% in the no nutritional risk group and the nutritional risk group, respectively, showing no statistically significant difference (*p* = 0.75). Alcohol consumers comprised 6.7% (21 cases) of the overall population, with proportions of 7.0 and 5.5% in the two groups, respectively, and no statistically significant difference (*p* > 0.99). Diabetic patients accounted for 7.7% (24 cases) of the total population, with proportions of 8.5 and 3.6% in the two groups, but the difference was not statistically significant (*p* = 0.27). Hypertensive patients comprised 27% (85 cases) of the total population, with proportions of 27 and 29% in the two groups, showing no statistically significant difference (*p* = 0.72). Patients with kidney disease accounted for 5.8% (18 cases) of the total population, with proportions of 5.8 and 5.5% in the two groups, respectively, and no statistically significant difference (*p* > 0.99). Cardiovascular disease patients comprised 14% (44 cases) of the total population, with proportions of 13 and 18% in the two groups, showing no statistically significant difference (*p* = 0.33). Cerebrovascular disease patients accounted for 12% (36 cases) of the total population, with proportions of 11 and 15% in the two groups, but the difference was not statistically significant (*p* = 0.44).

Multiple indicators, including systolic blood pressure (SBP), diastolic blood pressure (DBP), and creatinine (Cr), showed no statistically significant differences between the two groups. However, some indicators, such as the partial pressure of oxygen difference (PO2_d), exhibited a statistically significant difference (*p* = 0.045) (Table 1).

**Table 1:** Basic characteristics of the study subjects.

Variable	Overall	Non nutritional risk	Nutritional risk	*p*-value^2^
	*N* = 313^1^	*N* = 258^1^	*N* = 55^1^	
Gender				0.92
Male	203 (65%)	167 (65%)	36 (65%)	
Female	110 (35%)	91 (35%)	19 (35%)	
Age	74 (10)	74 (10)	75 (11)	0.47
WBC	7.7 (6.1, 9.9)	7.7 (6.1, 9.9)	8.4 (5.8, 10.5)	0.95
Neutrophils	5.40 (4.07, 7.48)	5.38 (4.13, 7.45)	5.50 (3.82, 7.61)	0.99
lymphocyte	1.22 (0.80, 1.64)	1.19 (0.78, 1.64)	1.31 (1.00, 1.68)	0.15
Hemoglobin	130 (115, 141)	131 (117, 142)	123 (106, 133)	0.005
Platelet	220 (181, 279)	220 (181, 267)	249 (177, 318)	0.28
C_reactive_protein	3 (1, 8)	3 (1, 5)	8 (2, 22)	0.004
Procalcitonin	0.03 (0.02, 0.06)	0.03 (0.02, 0.06)	0.03 (0.02, 0.05)	0.56
PAB	182 (133, 225)	184 (136, 227)	159 (127, 210)	0.065
Alb	39.1 (35.3, 42.5)	39.2 (35.3, 42.6)	37.8 (34.7, 42.2)	0.29
BMI	20.3 (18.4, 23.3)	21.3 (19.2, 23.6)	16.9 (15.6, 17.7)	<0.001
Hospital_Days	6.00 (5.00, 8.00)	6.00 (5.00, 8.00)	6.00 (4.00, 7.00)	0.12
Smoke				0.75
Yes	228 (73%)	187 (72%)	41 (75%)	
No	85 (27%)	71 (28%)	14 (25%)	
Drink				>0.99
Yes	21 (6.7%)	18 (7.0%)	3 (5.5%)	
No	292 (93%)	240 (93%)	52 (95%)	
SBP	132 (121, 143)	132 (121, 144)	127 (121, 142)	0.33
DBP	80 (75, 86)	80 (75, 86)	80 (75, 86)	0.94
Cr	71 (60, 87)	70 (59, 88)	72 (60, 85)	>0.99
ALT	16 (12, 22)	16 (12, 22)	14 (11, 24)	0.5
AST	19 (16, 24)	19 (16, 24)	18 (15, 26)	0.47
TG	0.93 (0.69, 1.27)	0.94 (0.68, 1.27)	0.92 (0.69, 1.37)	0.88
GLU	5.65 (4.66, 7.98)	5.61 (4.74, 8.26)	6.26 (4.44, 7.49)	0.39
PCO2_d	0.00 (−0.23, 0.20)	0.00 (−0.20, 0.20)	0.00 (−0.58, 0.19)	0.47
P02_d	0.0 (−0.5, 0.8)	0.0 (−0.1, 0.9)	0.0 (−2.7, 0.0)	0.045
Ventilator				0.87
Yes	49 (16%)	40 (16%)	9 (16%)	
No	264 (84%)	218 (84%)	46 (84%)	
Diabetes				0.27
Yes	24 (7.7%)	22 (8.5%)	2 (3.6%)	
No	289 (92%)	236 (91%)	53 (96%)	
Hypertension				0.72
Yes	85 (27%)	69 (27%)	16 (29%)	
No	228 (73%)	189 (73%)	39 (71%)	
KD				>0.99
Yes	18 (5.8%)	15 (5.8%)	3 (5.5%)	
No	295 (94%)	243 (94%)	52 (95%)	
Cardiovascular diseases				0.33
Yes	44 (14%)	34 (13%)	10 (18%)	
No	269 (86%)	224 (87%)	45 (82%)	
Cerebrovascular diseases				0.44
Yes	36 (12%)	28 (11%)	8 (15%)	
No	277 (88%)	230 (89%)	47 (85%)	

1 Mean (sd) or Frequency (%) or Median (IQR).

2 Pearson’s Chi-squared test; Wilcoxon rank sum test; Fisher’s exact test.

### 3.2 Association between C-reactive protein and nutritional risk

This study investigated the association between C-reactive protein (CRP) and nutritional risk using three hierarchical models: Model 1 (crude), Model 2 (adjusted for sex, age, smoking, alcohol consumption, systolic/diastolic blood pressure), and Model 3 (further adjusted for respiratory failure, diabetes, hypertension, kidney disease, cardiovascular/cerebrovascular diseases, and oxygen partial pressure variation). Continuous CRP demonstrated significant nutritional risk associations across all models: Model 1 (OR = 1.007, 95%CI: 1–1.014, *p* = 0.0321), Model 2 (OR = 1.007, 95%CI: 1–1.014, *p* = 0.0483), and Model 3 (OR = 1.009, 95%CI: 1.001–1.016, *p* = 0.016). Quartile analysis using Q1 (<1.48 mg/L, 24.6%, *n* = 77) as reference revealed non-significant associations for Q2 (1.48–<3.41, 25.24%, *n* = 79) and Q3 (3.41–<8.36, 24.92%, *n* = 78), while Q4 (≥8.36, 25.24%, *n* = 79) showed significant risk elevation in Model 1 (OR = 2.657, 1.247–5.931, *p* = 0.0134), Model 2 (OR = 2.465, 1.144–5.553, *p* = 0.0242), and Model 3 (OR = 3.193, 1.407–7.625, *p* = 0.0068). Significant dose–response trends were observed across models (Model 1: *p* = 0.0039; Model 2: *p* = 0.0091; Model 3: *p* = 0.0022), confirming CRP-level-dependent nutritional risk escalation (Table 2).

**Table 2:** The associations of C-reactive protein related indicators between nutritional risk.

Characterisitic	Exposure cutoff	Case (%)	Model 1			Model 2			Model 3		
			OR	95%CI	*p*-value	OR	95%CI	*p*-value	OR	95%CI	*p*-value
C-reactive protein											
C-reactive protein continuous			1.007	(1,1.014)	0.0321	1.007	(1,1.014)	0.0483	1.009	(1.001,1.016)	0.016
C-reactive protein quantile											
Q1 (low)	< 1.48	77 (24.6%)	Ref	Ref		Ref	Ref		Ref	Ref	
Q2	1.48- < 3.41	79 (25.24%)	0.527	(0.186,1.39)	0.2045	0.536	(0.187,1.432)	0.2223	0.576	(0.196,1.578)	0.2922
Q3	3.41- < 8.36	78 (24.92%)	0.797	(0.316,1.971)	0.6225	0.731	(0.287,1.828)	0.503	0.897	(0.34,2.337)	0.8238
Q4 (high)	≥ 8.36	79 (25.24%)	2.657	(1.247,5.931)	0.0134	2.465	(1.144,5.553)	0.0242	3.193	(1.407,7.625)	0.0068
*p* for trend					0.0039			0.0091			0.0022

The model 1 was the crude model.

The model 2 was adjusted by Gender, Age, Smoke, Drink, SBP, DBP.

The model 3 was adjusted by Gender, Age, Smoke, Drink, SBP, DBP, respiratory failure, diabetes, hypertension, KD, Cardiovascular Diseases, Cerebrovascular Diseases, P02_d.

### 3.3 XGBoost-based machine learning analysis of nutritional risk-associated factors

**Figure 2:**
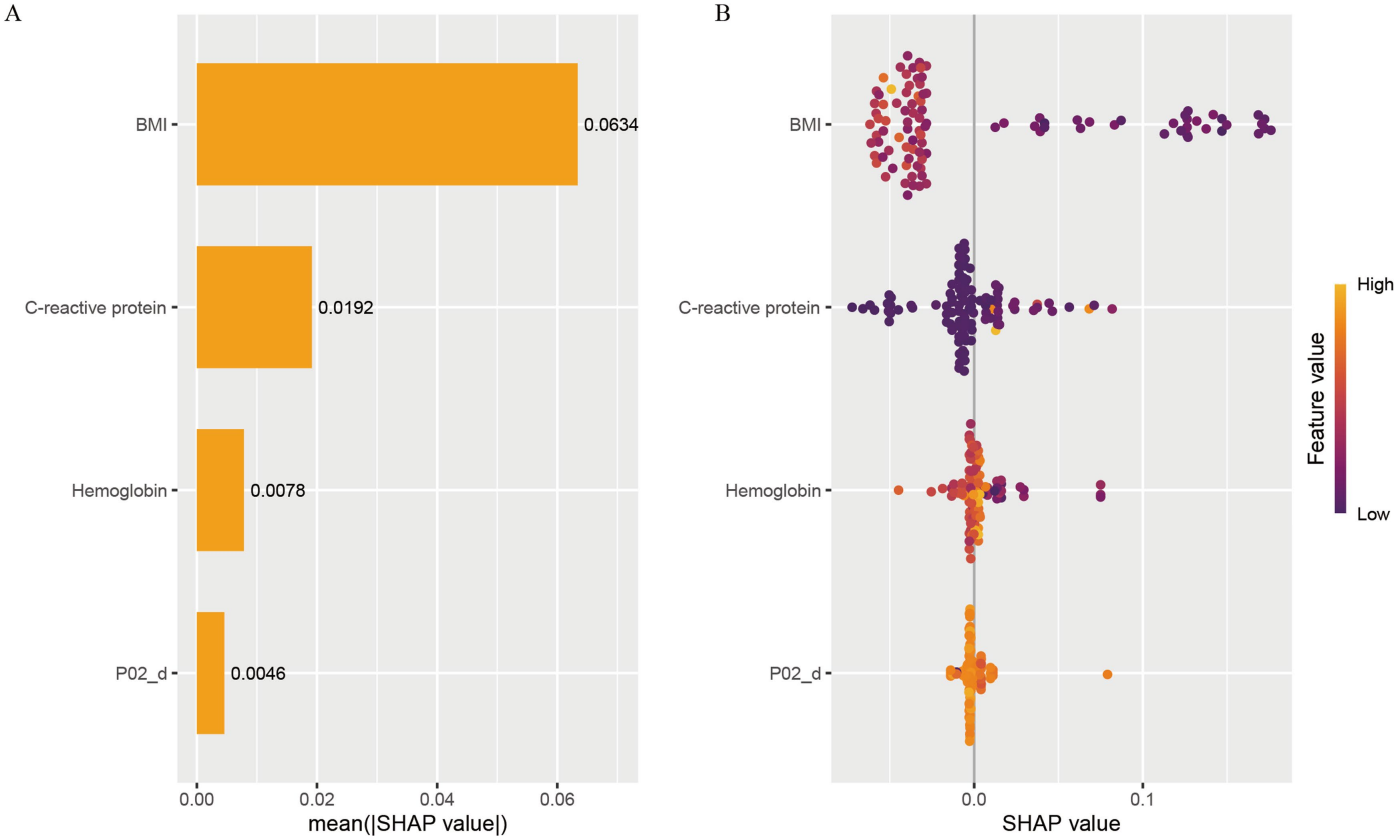
Illustrates the machine learning model through shap summary and dependency graphs PO2_d, changes in oxygen partial pressure. **(A)** SHAP tree diagram of Xgboost model characteristics. **(B)** Ranking of importance of Xgboost model features.

Figure 2 demonstrates the interpretability of the machine learning model through SHAP (SHapley Additive exPlanations) summary and dependency plots, elucidating feature-specific contributions to the XGBoost model.

The figure demonstrates key variables including BMI, C-reactive_protein, Hemoglobin, and P02_d, with their mean absolute SHAP values quantified as follows: BMI = 0.0634, C-reactive_protein = 0.0192, Hemoglobin = 0.0078, and P02_d = 0.0046.

The SHAP scatter plot illustrates the relationship between SHAP values and corresponding feature values for individual samples. For C-reactive protein (CRP), SHAP values ranged from −0.10 to 0.10, with color coding reflecting feature values (orange: high; purple: low). Purple data points (low CRP values) predominantly clustered in negative SHAP value regions. This distribution suggests that lower CRP feature values correlate with reduced model-predicted nutritional risk probabilities, while elevated CRP levels may indicate increased nutritional risk susceptibility.

### 3.4 Restricted cubic splines (RCS) curves of nutritional risk with C-reactive protein and subgroups

**Figure 3:**
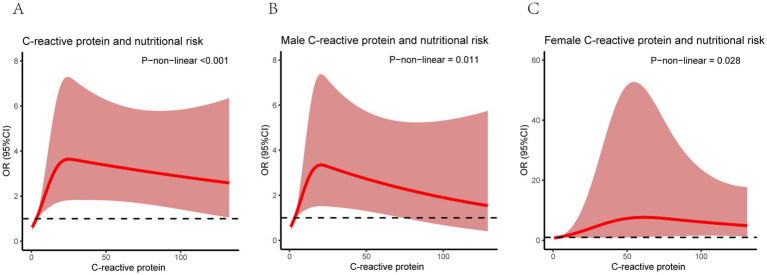
RCS curve of the relationship between C-reactive protein and nutritional risk. **(a)** all patients, **(b)** male patients, and **(c)** female patients.variance: gender, age, smoke, drink, SBP, DBP, respiratory failure, diabetes, hypertension, KD, cardiovascular diseases, cerebrovascular diseases, PO2_d.

Restricted cubic splines (RCS) were employed to investigate the relationship between C-reactive protein (CRP) and nutritional risk, with adjustment for all relevant covariates. A statistically significant nonlinear association was observed between CRP and nutritional risk (*p* for nonlinearity <0.001, Figure 3A). Furthermore, nonlinear relationships remained significant in both male (*p* for nonlinearity = 0.011, Figure 3B) and female subgroups (*p* for nonlinearity = 0.028, Figure 3C).

### 3.5 Association between C-reactive protein levels and nutritional risk across baseline characteristic subgroups

**Figure 4:**
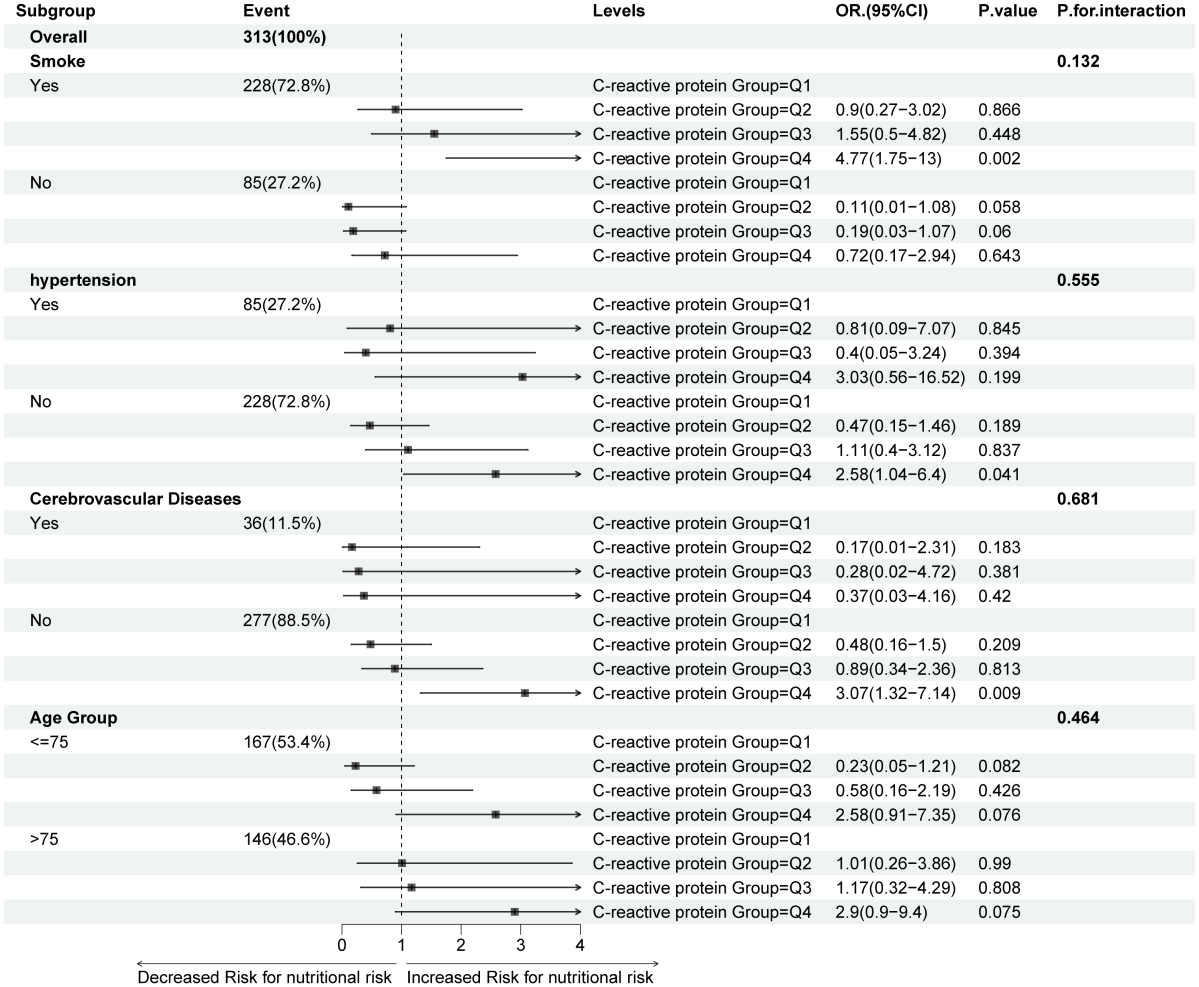
Relationship between baseline characteristic subgroups’ C-reactive protein levels and nutritional.

This study analyzed associations between C-reactive protein (CRP) quartiles (Q1-Q4) and nutritional risk across subgroups (smoking, hypertension, cerebrovascular disease, age) among 313 cases. In the smoking subgroup (*n* = 228, 72.8%), Q4 CRP demonstrated significantly elevated nutritional risk (OR = 4.77, 95%CI: 1.75–13, *p* = 0.002), while non-smokers (*n* = 85, 27.2%) showed only marginal Q2-Q3 trends (interaction *p* = 0.132). Hypertensive patients (*n* = 85, 27.2%) exhibited null associations, whereas non-hypertensive individuals (*n* = 228, 72.8%) demonstrated potential Q4 association (OR = 2.58, 1.04–6.4, *p* = 0.041; interaction *p* = 0.555). Cerebrovascular disease patients (*n* = 36, 11.5%) showed no significance, contrasting with non-cerebrovascular counterparts (*n* = 277, 88.5%) where Q4 reached significance (OR = 3.07, 1.32–7.14, *p* = 0.009; interaction *p* = 0.681). Age-stratified analyses revealed non-significant trends (≤75 years: *n* = 167, 53.4%; >75 years: *n* = 146, 46.6%; interaction *p* = 0.464). Collectively, elevated CRP (Q4) showed suggestive nutritional risk associations in smoking, non-hypertensive, and non-cerebrovascular subgroups without significant interaction heterogeneity (Figure 4).

## 4 Discussion

Previous studies have predominantly focused on nutritional risk in stable COPD patients (8, 9), whereas this investigation enrolled hospitalized individuals with AECOPD, where infection-induced metabolic surge amplifies nutritional demand-consumption imbalance. This study represents the first examination of the association between CRP levels and nutritional risk.

(1)  Significant differences were observed between nutritional risk and non-risk groups in BMI, hemoglobin, C-reactive protein (CRP), and oxygen partial pressure variation (all *p* < 0.05). These disparities suggest potential associations between nutritional risk status and hemoglobin levels, CRP expression, body composition, and respiratory function in this cohort. The nutritional risk group exhibited decreased hemoglobin levels (120 vs. 129), which may induce tissue hypoxia, subsequently activating inflammatory cells (10) and elevating CRP levels (26 vs. 14). Concurrently, hypoxia-mediated impairment of nutrient absorption/ utilization could exacerbate nutritional risk, warranting future investigation into their dynamic interplay. Notably, the nutritional risk group demonstrated significantly lower BMI (17.0 vs. 21.7), consistent with previous studies (8), supporting low BMI as a practical clinical indicator for nutritional risk stratification (11).

The elevated CRP and reduced hemoglobin levels further indicate a vicious cycle between chronic inflammation and metabolic dysregulation: pro-inflammatory cytokines (e.g., IL-6, TNF-*α*) exacerbate malnutrition through muscle catabolism and appetite suppression (12), while malnutrition impairs immunomodulatory capacity, perpetuating inflammatory responses. The intergroup oxygen partial pressure variation suggests potential interactions between impaired respiratory function and nutritional risk, emphasizing the necessity for integrated nutritional-respiratory care strategies in pulmonary disease management.

(2)  Across three progressively adjusted models, we demonstrated that each unit increase in C-reactive protein (CRP) as a continuous variable elevated nutritional risk probability (Model 3 OR = 1.009, 95%CI: 1.001–1.016, *p* = 0.016). Quartile analysis corroborated this finding, with only the highest quartile (Q4: CRP ≥ 8.36) showing significant association (Model 3 OR = 3.193, *p* = 0.0068), indicating a distinct threshold effect. Significant dose–response relationships persisted across all models (*p* for trend <0.01), supporting CRP’s utility as a sensitive biomarker for nutritional risk stratification.
(3)  The SHAP analysis of the XGBoost model demonstrated that BMI exhibited the highest contribution to nutritional risk prediction, substantially exceeding C-reactive protein (CRP), hemoglobin, and oxygen partial pressure variation (P02_d). This finding complements the logistic regression results: while traditional statistical models emphasized the independent effect of CRP, machine learning highlighted BMI’s pivotal role in comprehensive prediction. The prominence of BMI in SHAP analysis aligns with clinical practice prioritizing nutritional interventions based on stable-phase BMI levels. Future studies should incorporate longitudinal data to clarify BMI-CRP relationships during acute and recovery phases. Notably, despite CRP’s lower SHAP values, it revealed significant nonlinear associations in restricted cubic spline (RCS) curves (P for nonlinearity <0.001 in the overall population), a pattern maintained in both male (*p* = 0.011) and female (*p* = 0.028) subgroups. These findings suggest the necessity of causal inference methods to elucidate dynamic inflammation-nutrition interactions. Hemoglobin showed a mean SHAP value of 0.0078, while P02_d (defined as the difference between post-treatment and admission PaO2) displayed a mean SHAP value of 0.0046, indicating minimal predictive contributions. The inclusion of P02_d implies potential links between inflammation-mediated pulmonary dysfunction, metabolic hypoxia, and malnutrition.
(4)  This study underscores the necessity of routine CRP monitoring in clinical practice to facilitate early identification of high nutritional risk patients, potentially reducing hospitalization duration and improving survival (9, 10). While initiating early nutritional interventions for patients with CRP ≥ 8.36 mg/L is recommended, several limitations warrant consideration. First, CRP’s inherent limitations as an inflammatory marker: despite widespread use, its elevation may stem from infections, autoimmune disorders, or other inflammatory states beyond nutritional risk. Second, the retrospective observational design precludes causal determination – whether elevated CRP directly induces nutritional risk, malnutrition exacerbates inflammation, or both share common underlying factors remains unclear.

Clinical implementation complexities demand attention: patient heterogeneity, disease progression, and comorbidities may necessitate nutritional support even when CRP falls below 8.36 mg/L, while CRP-elevated patients with stable nutrition might require comprehensive evaluation before intervention. Personalized strategies integrating individual clinical profiles are essential for optimizing intervention applicability.

Data limitations from medical records, particularly incomplete dietary documentation, could compromise NRS-2002 accuracy and CRP-nutrition risk assessments. Future prospective multicenter cohorts should enroll diverse COPD patients with acute lower respiratory infections across severity strata, employing standardized CRP assays alongside inflammatory mediators (IL-6, TNF-*α*). Multivariable modeling could elucidate composite inflammatory impacts on nutritional risk, providing more definitive guidance for clinical management.

(5)  In AECOPD, inflammation serves not only as a core pathological feature of COPD and its comorbidities but also significantly impacts nutritional status. Substantial evidence indicates that inflammatory responses alter systemic metabolism, disrupting nutrient intake, absorption, and utilization, thereby precipitating malnutrition. Pro-inflammatory mediators, including tumor necrosis factor-alpha (TNF-*α*) and interleukin-6 (IL-6), suppress appetite via central nervous system modulation, reducing dietary intake and exacerbating nutritional deficits (13). Future investigations should establish longitudinal monitoring frameworks integrating CRP, IL-6, and TNF-α to disrupt the “inflammation-malnutrition” vicious cycle and improve long-term outcomes.

COPD studies demonstrate strong correlations between elevated inflammatory markers (e.g., CRP) and appetite suppression/reduced food intake (14), suggesting inflammation as a critical driver of malnutrition in this population (15, 16). Furthermore, inflammation perturbs nutrient metabolism and utilization. Hypermetabolic states induced by inflammation increase basal energy expenditure, amplifying malnutrition risk (17). In COPD, chronic inflammation accelerates proteolysis and lipolysis, compromising physiological homeostasis under nutritional deprivation (18).

The inflammation-nutrition interplay exhibits context-dependent characteristics. AECOPD intensify inflammatory cascades, accelerating nutritional deterioration. The Nutritional Risk Screening 2002 (NRS2002) proves valuable for assessing COPD patients, with studies revealing significant CRP-nutrition risk correlations (higher risk associated with elevated CRP (19, 20)), reflecting chronic inflammation’s dual impact on nutritional status and clinical trajectories (21, 22). Clinically, CRP-integrated screening enables precise nutritional risk identification in patients with AECOPD, facilitating timely interventions critical for mitigating inflammation and optimizing holistic health outcomes.

## 5 Conclusion

This study highlights the significant association between CRP levels and nutritional risk in patients with AECOPD, providing evidence-based support for nutritional risk assessment and early intervention in this population.

## Data Availability

The datasets presented in this article are not readily available because the data can only be used for medical research purposes. Requests to access the datasets should be directed to ganranke123456@21cn.com.
